# A Target Tracking Method Based on a Pyramid Channel Attention Mechanism

**DOI:** 10.3390/s25103214

**Published:** 2025-05-20

**Authors:** Dongxuan Zhao, Yunsong Li, Jiaxing Li, Xiping Duan, Ning Ma, Yuhe Wang

**Affiliations:** School of Computer Science and Information Engineering, Harbin Normal University, No. 1 Shida Road, Limin Economic Development Zone, Harbin 150025, China; devin.wit.dx@gmail.com (D.Z.); 15776840972@163.com (Y.L.); 2024300712@stu.hrbnu.edu.cn (J.L.); maning@hrbnu.edu.cn (N.M.); cs2008wyh@163.com (Y.W.)

**Keywords:** target tracking, deep learning, transformer, feature extraction, feature fusion

## Abstract

To enhance the tracking performance of transformer-based trackers in complex scenes, we propose a novel visual object tracking method that incorporates three key components: a pyramid channel attention mechanism, a hierarchical cross-attention structure, and an attention-guided multi-layer perceptron. The pyramid channel attention mechanism dynamically enhances informative feature channels across different scales, while the hierarchical cross-attention structure facilitates effective feature interaction. The attention-guided multi-layer perceptron introduces nonlinear transformations under attention guidance to improve feature representation. Experimental results on benchmark datasets demonstrate the superior performance of the proposed method.

## 1. Introduction

In today’s information-driven era, the rapid evolution of artificial intelligence (AI) technology has been a key driver of continuous advancements across the scientific and technological landscape, with particularly remarkable strides made in the field of computer vision. As an interdisciplinary research domain, computer vision is dedicated to empowering computer systems with the ability to parse and comprehend visual information, a capability whose significance is self-evident. The ongoing maturation of deep learning technology has propelled computer vision to achieve groundbreaking progress in several key tasks, including image recognition [1], image classification [2], and object tracking [3]. These advancements have delivered more efficient and precise solutions tailored to a wide range of application scenarios.

Object tracking is a key research issue in computer vision, with great theoretical significance and wide practical applications. Its main aim is to accurately identify and continuously track specific objects in video streams. This involves analyzing not only the information from the current frame but also the object’s historical information from previous frames, such as position, speed, and shape. Through comprehensive analysis and modeling of this information, object tracking predicts the object’s location in the current frame. In contrast, object detection [4] is closely related to, yet distinct from, object tracking and mainly deals with static information from single images or videos, determining the existence, location, and category of objects. It focuses on the presence and position of objects in the current frame, with less consideration of historical information. The capabilities of object tracking are crucial to numerous fields, such as intelligent video surveillance [5], human–computer interaction [6], and intelligent transportation systems.

The evolution of object tracking technology can be broadly categorized into two main stages: traditional machine learning methods and deep learning-based approaches. In the early stages, traditional machine learning methods demonstrated their effectiveness in specific application scenarios. However, their performance was notably constrained in complex environments characterized by dynamic background changes and fast-moving target tracking. The advent of deep learning, particularly the development of models such as convolutional neural networks (CNNs) [7], has marked a qualitative leap in the accuracy and robustness of object tracking, as well as other computer vision tasks [8]. These deep learning models, leveraging their powerful feature extraction and representation learning capabilities, have significantly enhanced various computer vision tasks, such as identification, building façade analysis [9], construction site monitoring [10], image augmentation [11], and object detection [12,13,14,15]. Despite these advancements, deep learning methods still exhibit limitations in handling long sequence data and capturing global dependencies, prompting researchers to explore more efficient algorithms to further elevate performance.

Recently, the transformative progress of the transformer architecture [16] in the field of deep learning has garnered widespread attention. By incorporating advanced technologies such as self-attention mechanisms and position encoding, this architecture has effectively captured long-range dependencies in sequence data, introducing revolutionary new ideas and methods in the field of object tracking. In particular, the transformer architecture’s exceptional global information processing capabilities have greatly improved the accuracy of target identification and localization in object tracking tasks. However, despite the significant progress made by the transformer architecture in this domain, tracking models based on this architecture still exhibit some shortcomings in two critical aspects: feature extraction and feature fusion.

For example, the TransT model [17] utilizes a transformer-based architecture to effectively integrate the global information of the target and search area through nonlinear semantic fusion and long-range feature association. While this approach has demonstrated excellent performance on multiple tracking datasets, the TransT model still encounters challenges when processing high-dimensional visual data. Specifically, it struggles with extracting and integrating deep, multi-scale feature information, as well as optimizing the feature fusion process to more accurately represent the global and local characteristics of the target.

To address the challenges of feature extraction and feature fusion, this study proposes an innovative object tracking method based on a pyramid channel attention mechanism. By introducing this mechanism to improve the feature extraction network and incorporating hierarchical cross-attention mechanisms and attention-based multi-layer perceptrons (aMLPs) [18] into the feature fusion network, the proposed method effectively captures target information at different scales. This enhances the robustness and tracking accuracy of the algorithm. These innovative improvements not only bolster the algorithm’s adaptability in handling complex scenarios such as occlusion and deformation but also significantly elevate the overall performance of the object tracking algorithm. The innovative aspects of this research primarily include the following:

(1) The introduction of a pyramid channel attention mechanism and its integration into the feature extraction network for object tracking. This approach realizes dynamic weight adjustment on the feature map in the channel dimension by incorporating a pyramid channel attention module within the residual network structure of the feature extraction network. Specifically, the module enhances feature channels that significantly contribute to object tracking while suppressing those with fewer contributions, thereby optimizing the quality of the feature representation. With the improved feature extraction network, this method not only enhances the quality of feature representation but also increases the efficiency of utilizing multi-scale information.
(2) The proposal of a hierarchical cross-attention mechanism and its integration into the feature fusion network for object tracking. This method facilitates feature interaction at different levels by introducing a hierarchical cross-attention mechanism into the feature fusion network, promoting the exchange of information across various scales and regions, thereby effectively enhancing the expressive power of the features.
(3) The adoption of an attention-based multi-layer perceptron (aMLP) in the feature fusion network for object tracking, replacing the traditional multi-layer perceptron. The aMLP further improves the discriminative power of features through attention-guided nonlinear transformations.

These innovative improvements significantly enhance the model’s accuracy and flexibility in target localization and tracking. Experimental results demonstrate that the improved method achieves an average overlap rate of 0.625 on the GOT-10k [19] dataset and a success rate of 0.696 on the OTB-100 [20] dataset, validating its effectiveness.

Following experimental validation, tracking methods based on pyramid channel attention mechanisms have proven to be significantly effective in enhancing the precision of object tracking. This study not only enriches the technical methods of object tracking but also provides new perspectives and directions for future research in the field of object tracking.

## 2. Related Works

The advent of Siamese networks has garnered widespread attention in the field of object tracking. Pioneering works such as SiamFC [21] and SiNT [22] have employed Siamese network architectures, leveraging weight-sharing dual-branch convolutional neural networks for feature extraction from template and search region images, ensuring consistency in feature representation. Through similarity assessment modules and cross-correlation operations, peak pixel values in the generated response maps indicate the precise location of the target within the search region, further enhancing positional accuracy through non-maximum suppression (NMS).

To enhance tracking performance, various improvements have been made to Siamese network tracking models. SiamRPN [23] uses region proposal networks to generate candidate boxes, effectively addressing target occlusion and deformation issues. SiamMask [24] incorporates segmentation branches to achieve real-time object segmentation, enhancing robustness. DaSiamRPN [25] utilizes attention mechanisms to enhance feature representation. Additionally, SiamBAN [26] and SiamCAR [27] use boundary-aware branches and context-aware modules, respectively, to optimize predictions. Furthermore, the optimization of loss functions and training data, such as binary response map supervision in SiamDW [28] and joint training on large-scale datasets in SiamFC++ [29], further strengthens the model’s feature representation and generalization capabilities.

The typical framework of Siamese network-based object tracking models comprises three parts: a feature extraction network, a feature fusion network, and a prediction head, enhancing its effectiveness in object tracking tasks. Leveraging this architecture, we propose an efficient and robust tracking method by incorporating a pyramid channel attention mechanism to optimize the feature extraction network and improve the connectivity of the feature fusion network.

In the realm of object tracking, the deep residual network architecture of the Residual Network (ResNet) [29] has revolutionized feature extraction by offering a groundbreaking approach. ResNet effectively mitigates the degradation problem often encountered when training deep networks, leveraging its innovative residual learning framework to enhance performance through increased network depth. This unique characteristic bestows ResNet with stable and robust feature representations, making it particularly adept at handling complex scenarios, such as variations in lighting conditions, occlusion, and deformation, which are common challenges in object tracking tasks.

However, the increased depth of ResNet, while yielding significant performance gains, also presents notable challenges. The complexity of model training escalates, and the demand for computational resources intensifies. To address these challenges, researchers have devised various optimization strategies. For example, the introduction of attention mechanisms, such as the Squeeze-and-Excitation (SE) module in SENet [30], allows networks to focus more intently on crucial target features, thereby significantly improving tracking accuracy. Moreover, optimizing training datasets and loss functions, such as utilizing large-scale datasets and designing more sophisticated loss functions, further enhances the generalization capability and robustness of ResNet.

In summary, ResNet’s deep residual network architecture has proven to be a powerful tool in object tracking, offering enhanced feature extraction and robustness. Despite the challenges posed by its depth, ongoing research and optimization strategies continue to unlock its full potential, making it an indispensable asset in modern object tracking applications.

The introduction of the transformer [31] architecture represents a significant leap in computer vision’s object tracking tasks. Initially proposed by Vaswani et al. for machine translation, this architecture consists of encoder–decoder components based on self-attention mechanisms. The advantages of self-attention lie in its effective integration of global information within the model while significantly reducing inductive biases, a trait corroborated across various domains.

In the realm of computer vision, the Vision Transformer (ViT) [32] and Detection Transformer (DETR) [33] represent pioneering applications of the transformer architecture in visual tasks, showcasing its potential in image classification and object detection. The transformer, with its self-attention mechanism, can capture complex relationships between different frames in video sequences. This provides a more comprehensive and in-depth understanding of spatiotemporal features, offering a new perspective for the development of object tracking technology.

In practical object tracking applications, the combination of transformer models and Siamese network structures has made it possible to build more powerful tracking frameworks. For example, the TransT algorithm integrates transformer layers into Siamese networks. Using the self-attention mechanism of transformers, it effectively fuses target features with search region features, significantly enhancing the model’s robustness for long-term object tracking. Similarly, the SwinTrack [34] algorithm uses a transformer for feature extraction and fusion. By enabling comprehensive interaction between target objects and search regions, it achieves precise object tracking.

However, despite showing significant potential in object tracking tasks, transformer models still face several challenges in feature extraction and fusion. For instance, the TransT and STARK [35] models use the ResNet architecture in the feature extraction stage. When processing images, this architecture may fail to fully capture multi-level and local features, limiting the model’s sensitivity to target details. In addition, the feature fusion process faces issues that require further research, such as more effectively integrating feature information from different levels and addressing the problem of information loss during feature fusion.

These challenges can lead to performance degradation of tracking models in complex scenarios, such as occlusion and background clutter. Therefore, future research directions should focus on addressing these challenges. This can be achieved by improving feature extraction networks, introducing architectures that can capture richer feature information, and designing more refined feature fusion strategies to enhance the robustness and accuracy of models in complex environments. Meanwhile, exploring the use of attention mechanisms to optimize the feature fusion process is necessary.

## 3. Method

In modern Siamese network architectures for object tracking, the task is typically decomposed into three main stages: feature extraction, feature fusion, and target prediction. However, these architectures face several challenges in practical applications. First, in the feature extraction stage, traditional approaches relying on features from the final layers overlook the importance of multi-scale features, thereby limiting the capability to handle complex scenes. Second, in the feature fusion stage, existing transformer-based models fail to effectively optimize information flow, resulting in inadequate interaction and discriminative power among features. To address these issues, this study proposes a novel, efficient, and robust object tracking method.

### 3.1. Overall Architecture

**Figure 1:**
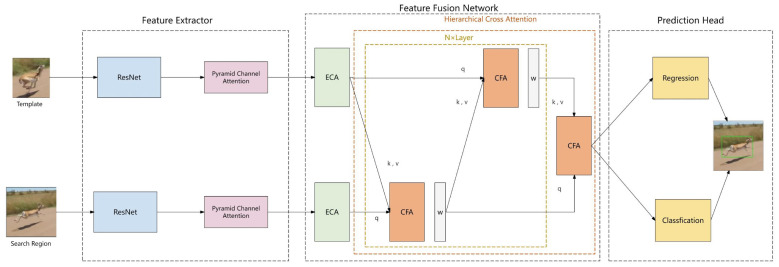
Overall architecture of our object tracking model.

In this research, we present an innovative pyramid channel attention mechanism-based object tracking method and construct an integrated framework. The framework is composed of three key components: a feature extraction network, a feature fusion network, and a prediction head. These components correspond to the three core stages of the object tracking process: feature extraction, feature fusion, and target prediction. For the sake of the subsequent discussion, we name our model the PCA-T model, and its architectural details are illustrated in Figure 1.

The feature extraction network serves as the cornerstone of the algorithm, with its primary task being the extraction of key features from the template and search images. This study employs a Siamese network architecture, which includes two sub-networks that share weights, each dedicated to processing the template and search image, respectively. This design enables the network to efficiently learn the correlation between the two images and provide precise feature information for subsequent target localization. Furthermore, to enhance the discriminatory power and expressive capacity of the features, we integrate a pyramid channel attention mechanism (PCAM) based on the Siamese network. This mechanism enhances the network’s adaptability to changes in target size and morphological differences by constructing multi-scale feature representations and strengthening the responses of key features, thereby significantly improving the accuracy and efficiency of feature extraction.

As the core of the algorithm, the feature fusion network is responsible for efficiently integrating the extracted features. In this study, the feature fusion network is based on the transformer architecture and utilizes self-attention and cross-attention mechanisms to enhance the interaction between features. The self-attention mechanism allows the model to capture the internal, detailed associations among features, while the cross-attention mechanism strengthens the interaction between the template and search image features. In addition, the introduced hierarchical cross-attention mechanism enables the model to more accurately identify the differences between the target and the background, significantly enhancing tracking ability in complex environments. To further enhance the model’s nonlinear modeling capability, we replace the multi-layer perceptron (MLP) in the feature fusion network with an adaptive multi-layer perceptron (aMLP). This fusion strategy not only improves the model’s adaptability under conditions of deformation and occlusion but also significantly enhances tracking accuracy and robustness.

As the final output stage of the model, the prediction head has the primary task of converting the fused features into specific tracking results. Through classification and regression analysis, the model can accurately predict the location and size of the target in the search image. Classification analysis is used to determine whether the feature points belong to the target area, while regression analysis is used to estimate the offset of the feature points from the center of the target. This design ensures the accuracy and reliability of the model in target localization, providing a solid foundation for achieving efficient and stable object tracking.

### 3.2. Pyramid Channel Attention Mechanism

**Figure 2:**
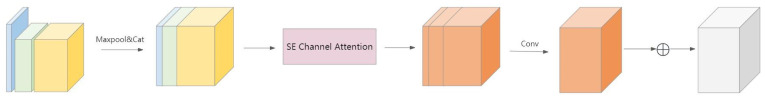
Pyramid channel attention mechanism.

We propose a channel attention mechanism based on a pyramid structure to augment feature map expressiveness and multi-scale information fusion for target tracking tasks. This mechanism dynamically learns the weight distribution across feature maps at various levels in the channel dimension, emphasizing pertinent channels for target tracking while suppressing irrelevant ones. The rationale behind our design rests on two principles: first, feature maps at distinct levels exhibit varying semantic and detail levels, warranting adaptive channel importance adjustments to enhance feature map effectiveness and robustness. Second, complementary information exists between feature maps of different levels; thus, effective fusion can amplify multi-scale information. Figure 2 illustrates the implementation of the PCAM mechanism.

The pyramid channel attention mechanism comprises the steps outlined below.

First, the feature map (F1) of ResNet’s Layer 1 undergoes spatial resolution halving to align with Layer 2’s feature map (F2) and Layer 3’s feature map (F3), ensuring uniform spatial resolutions across different levels of feature maps, thereby facilitating subsequent channel attention computation and fusion.

Second, concatenating these three-layer feature maps along the channel dimension yields a tensor (C1 + C2 + C3) × H × W, where C1, C2, and C3 denote the channel counts of F1, F2, and F3, respectively, and H and W represent the spatial resolutions. This step amalgamates feature maps of diverse levels to enable channel attention calculation and fusion.

Third, this tensor is fed into an SE channel attention module, which learns a (C1 + C2 + C3)-dimensional vector signifying the importance of each channel. This vector is then multiplied element-wise with the concatenation tensor along the channel dimension to yield the weighted tensor. During this step, the channel attention mechanism weights each channel, reinforcing crucial channels while suppressing non-essential ones, thereby enhancing feature map expressiveness.

Fourth, a 1 × 1 convolution layer reduces the channel count of the weighted tensor to C3, and a residual connection with F3 is established to generate the final output feature map. This step fuses the attention-weighted feature map with the original, preserving original information while incorporating attention-derived information.

**Figure 3:**
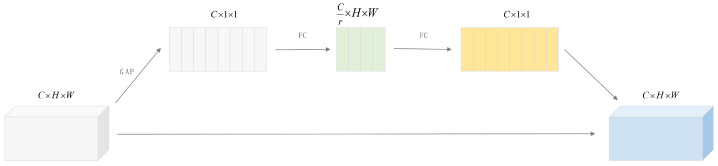
SE channel attention module.

Figure 3 depicts the SE channel attention module, which comprises two primary stages: extraction and excitation.

In the extraction stage, the input C × H × W dimensional tensor undergoes global average pooling to transform into a C × 1 × 1-dimensional vector, capturing each channel’s global response, thereby reducing computational cost and parameters while retaining global channel information.

The excitation phase constructs a bottleneck structure via two fully connected layers. The first layer reduces the C-dimensional vector’s dimension to C/r, where r is the scaling factor (default value: 16). After ReLU activation, the second layer restores the vector’s dimension to C and outputs channel attention weights via the Sigmoid activation function, confined to the range [0, 1].

This design reduces feature map dimensionality and computational load and increases nonlinearity.

Finally, the C-dimensional attention weight vector is multiplied element-wise with the input tensor along the channel dimension, yielding the output weighted tensor, thereby reinforcing important channels, suppressing unimportant ones, and enhancing feature map expressiveness.

In summary, the proposed pyramid-structured channel attention mechanism adaptively allocates channel weights across feature maps of different levels, effectively emphasizing pertinent channels and inhibiting irrelevant ones, thereby enhancing feature map semantics and detail levels and furnishing a robust foundation for enhanced tracking task performance, particularly in complex scenarios.

### 3.3. Hierarchical Cross-Attention Structure

In this investigation, we introduce a novel hierarchical cross-attention (HCA) framework designed to address the challenges inherent in target tracking paradigms. This framework is predicated on the enhancement of both the computational throughput and the tracking algorithm’s efficacy by refining the process of feature amalgamation.

**Figure 4:**
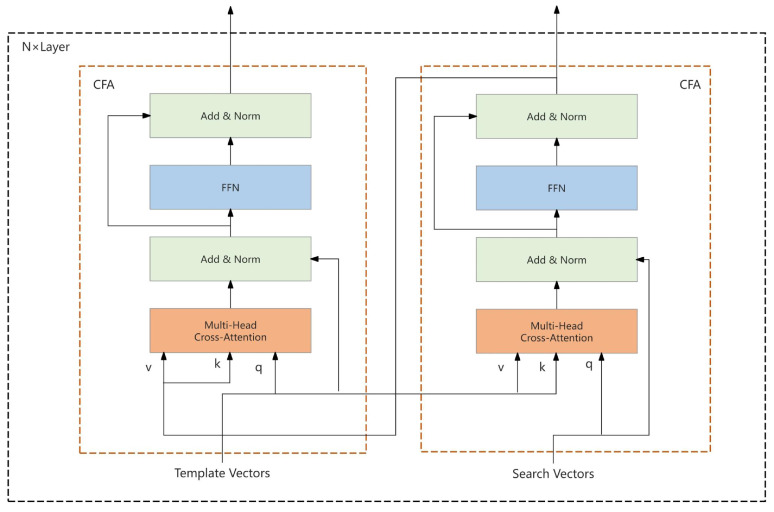
Connectivity of the cross-attention mechanism.

At the heart of the HCA framework is a multi-tiered cross-attention mechanism, which leverages the output of the cross-feature aggregation (CFA) module from the search area branch as the key input for the CFA module of the template branch, thereby establishing hierarchical inter-branch connectivity. As depicted in Figure 4, this connectivity schema delineates the interplay between the branches.

This strategic interconnection effectively mitigates superfluous computations within the framework’s infrastructure and facilitates the utilization of the search region’s rich target information by the template features, culminating in more precise feature integration. Furthermore, an adjustable weight parameter, denoted as w, is incorporated into each CFA module to adaptively modulate the weighting of feature channels, thus augmenting the flexibility and efficiency of the feature integration process.

Within the HCA framework, the features extracted from the search area and template branches are represented distinctly. These feature maps are integrated through the interplay of the cross-attention modules, with the output $F_{cfa_{s}}$ of the search area branch derived from the interaction between $F_{s}$ and $F_{t}$. The mathematical formulation of this process is articulated in Equation (1):

(1) $$F_{cfa_{s}}=w\cdot \mathrm{CFA}\left(F_{s},F_{t},F_{t}\right)$$

where *w* represents the learnable weight parameter that adaptively adjusts the ratio of the feature channel weights. Subsequently, the CFA module of the template region branch employs $F_{t}$ and $F_{cfa_{s}}$ to compute the output $F_{cfa_{t}}$ of the second CFA module, as articulated in Equation (2):

(2) $$F_{cfa_{t}}=w\cdot CFA\left(F_{t},F_{cfa_{s}},F_{cfa_{s}}\right)$$

Employing this iterative approach, the model refines the information flux and feature integration in a sequential manner by stacking multiple CFA modules. The feature map, post-multi-layer CFA processing, is then funneled into the final CFA module to yield the ultimate output $F_{f}inal$, as delineated in Equation (3):

(3) $$F_{final}=w\cdot CFA\left(F_{cfa_{s}},F_{cfa_{t}},F_{cfa_{t}}\right)$$

**Figure 5:**
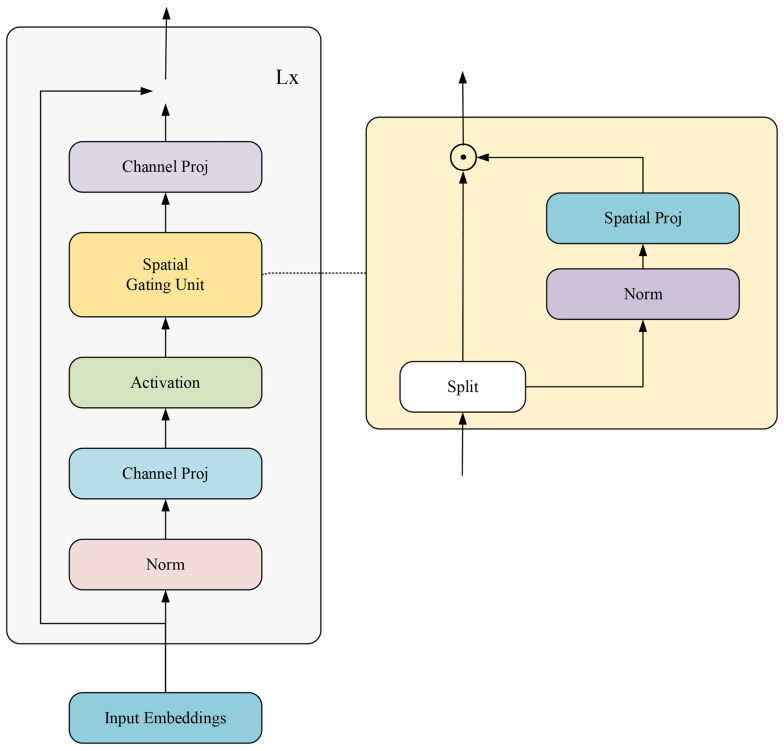
Structure diagram of the basic gMLP module.

**Figure 6:**
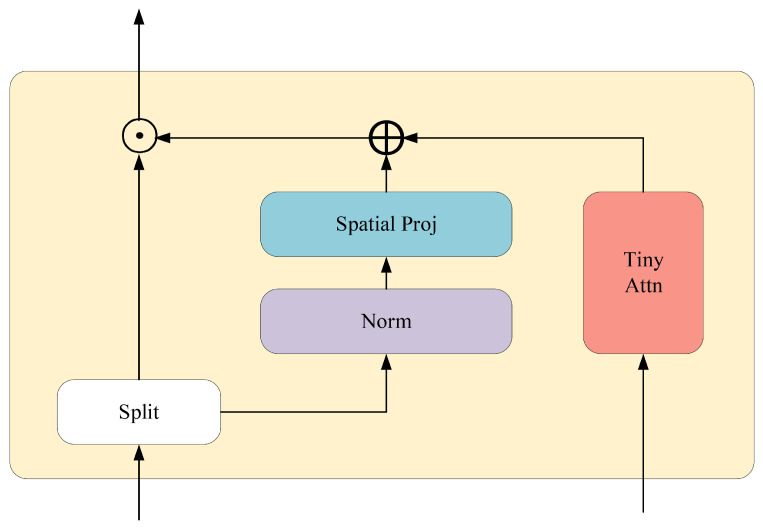
Structure diagram of the gating unit of aMLP.

**Figure 7:**
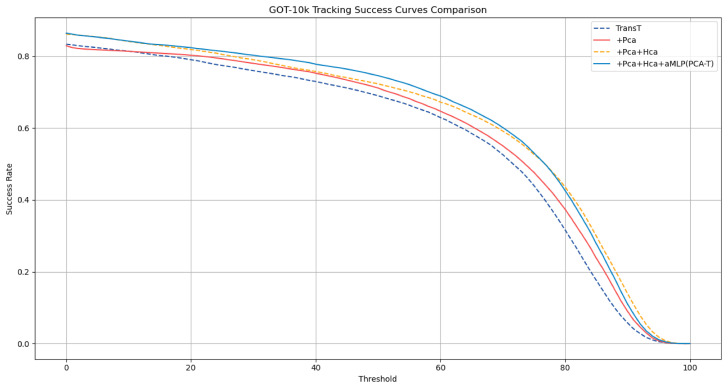
Success rate curves of various models in the ablation experiment on the GOT-10k dataset.

As illustrated in Figure 3, Figure 4, Figure 5, Figure 6 and Figure 7, the connectivity modes of the HCA mechanism are purposefully structured to optimize information flux through hierarchical linkages. The primary function of this architecture is to bolster the execution of target tracking tasks. By enabling the template branch to directly harness the feature information from the search region branch, the HCA framework facilitates the template features to account for the dynamic variations within the search region during the integration process, thereby enhancing the precision of target localization and tracking. The stratified, interconnected cross-attention modules enable the use of more refined features for integration, and the model can capitalize on the features processed in prior layers to achieve further precision in each subsequent layer. By eschewing complex interlayer connections and redundant computational pathways, the HCA framework simplifies the model architecture, rendering it more tractable for implementation and maintenance. Consequently, the HCA mechanism stands as a significant advancement in the domain of target tracking, offering an efficacious solution for real-time target tracking endeavors.

### 3.4. Enhanced Feature Fusion Networks with aMLP

The feature fusion network was optimized by integrating an aMLP [2], replacing the conventional multi-layer perceptron (MLP). The MLP, a foundational feedforward neural network, processes data via a series of interconnected layers with neurons that apply nonlinear activation functions to the output from preceding layers. This architecture is adept at capturing complex feature interactions between templates and search areas, enhancing a model’s representational power and robustness in tracking tasks.

However, the MLP’s fully connected layers may not efficiently encode spatial relationships within visual data, particularly in tasks like object tracking. To address this, the gated multi-layer perceptron (gMLP) incorporates a spatial gating unit (SGU) to enable spatial feature relationship modeling. The gMLP’s design improves spatial data processing capabilities and computational efficiency by reducing the parameter count.

As illustrated in Figure 5, the gMLP architecture includes sub-layers, such as the SGU, fully connected layers, and normalization layers. The SGU modulates input signals through gated variables, facilitating nonlinear signal transformation. The gMLP preprocesses input sequences through embedded layers and normalization before spatial information is modeled by the SGU, culminating in output via linear transformation and residual connections. Despite the gMLP’s spatial relationship handling, it may fall short in capturing long-range dependencies compared to self-attention models. To mitigate this, the aMLP introduces an adaptive attention mechanism, enhancing the model’s ability to model long-range dependencies. As shown in Figure 6, the design of the aMLP allows dynamic adjustment of gated variable weights based on input data characteristics, enabling more flexible feature dependency learning.

The aMLP’s core concept is the adaptive integration of spatial and sequential information, providing a versatile framework for diverse data types. This approach is particularly beneficial for applications requiring efficient large-scale data processing. In our experiments, replacing the traditional MLP with the aMLP in the feature fusion network significantly improved tracking model performance. The aMLP’s adaptive gating mechanism offers enhanced flexibility, allowing dynamic feature processing based on hierarchical characteristics, thus effectively integrating features and bolstering the network’s robustness against complex backgrounds and interference. Moreover, the aMLP facilitates effective feature fusion across scales, enhancing the model’s adaptability and recognition capabilities.

In conclusion, the aMLP’s incorporation into the feature fusion network not only elevated model performance but also offered novel insights for future object tracking applications in complex environments.

## 4. Experiments

### 4.1. Experimental Parameters

The method proposed in this paper was validated through a series of experiments conducted on a high-performance server platform. The server was configured with the Linux operating system, equipped with an Intel® Xeon® (The manufacturer of the Intel® Xeon® processor is Intel Corporation, headquartered in Santa Clara, CA, USA) Platinum 8255C processor, and featured an NVIDIA Tesla V100SXM2 (The NVIDIA Tesla V100S SXM2 is manufactured by NVIDIA Corporation, headquartered in Santa Clara, CA, USA) graphics card with 32GB of video memory. The deep learning framework utilized for the experiments was PyTorch version 1.9.0, and the programming language used was Python version 3.8.10. This setup ensured the stability and efficiency of the experimental environment, providing a reliable foundation for the evaluation of the algorithm’s performance.

The GOT-10K [3] was used for model training, with AdamW used as the optimizer. During the training phase, each batch consisted of 20 samples, and the entire training cycle included 40 epochs, with each epoch consisting of 1000 training pairs. To reduce the risk of overfitting, the dropout rate was set to 0.1 in the experiments. The initial learning rate for the feature extraction network was set to 0.00001, while the initial learning rates for the other structural parameters were set to 0.0001.

Regarding data processing, the model performed standardization on the input data, with the mean values set to 0.485, 0.456, and 0.406, and the standard deviations set to 0.229, 0.224, and 0.225. The search region factor was set to 4.0, and the template region factor was set to 2.0. The search feature size was set to 32, and the template feature size was set to 16. The center jitter factor was set to 3 for the search and 0 for the template, while the scale jitter factors were set to 0.25 and 0, respectively.

### 4.2. Ablation Experiment

To substantiate the effectiveness of the proposed modules, this section conducts an ablation study and performs an in-depth analysis on both the GOT-10k [3] and OTB-100 [4] datasets. Commencing with the GOT-10k dataset, the TransT method was employed as the reference baseline before progressively integrating the pyramid channel attention mechanism, hierarchical cross-attention mechanism, and advanced multi-layer perceptron (aMLP). After each module integration, a stringent performance evaluation was conducted to verify the accuracy of the experimental outcomes. The success rate (SR) and average overlap (AO) of the model were compared on the GOT-10k dataset, and the ablation study results are presented in Table 1 and illustrated in Figure 7.

Introducing the pyramid channel attention mechanism into the baseline TransT model resulted in an increase in the AO value from 0.572 to 0.593, an increase in the SR at an overlap threshold of 0.5 (SR0.5) from 0.690 to 0.711, and an increase in the SR at an overlap threshold of 0.75 (SR0.75) from 0.440 to 0.476. Following the integration of the hierarchical cross-attention mechanism, the AO further increased to 0.619, the SR0.5 to 0.723, and the SR0.75 to 0.527. Ultimately, upon integrating the aMLP, the AO reached 0.625, with the SR0.5 at 0.746 and the SR0.75 at 0.530. These results indicate that the integration of each component significantly contributes to the performance enhancement of the tracking model.

**Figure 8:**
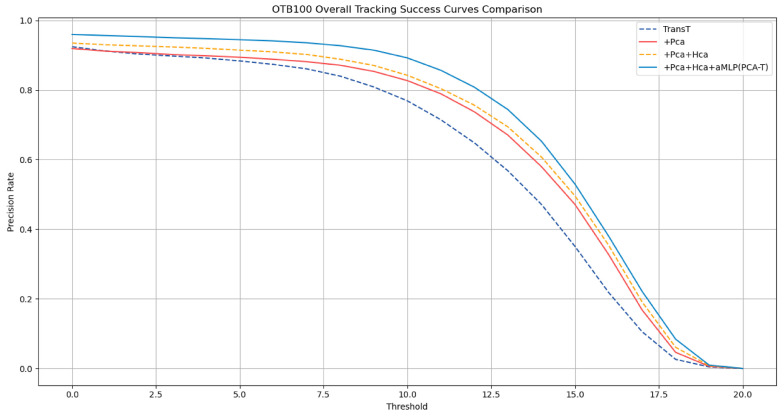
Success rate curves of various models in the ablation experiment on the OTB-100 dataset.

**Figure 9:**
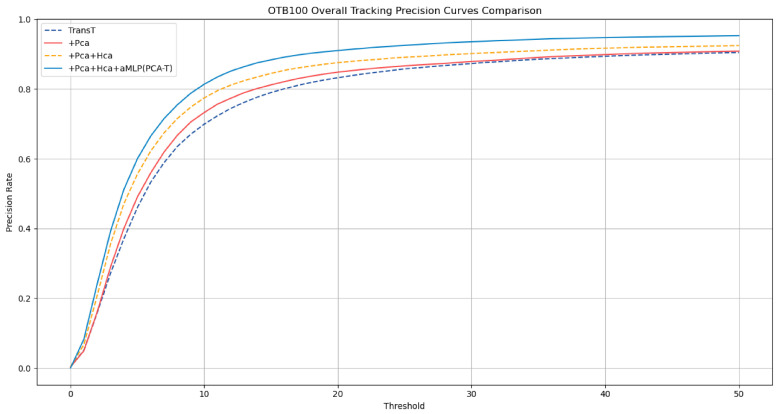
Precision rate curves of various models in the ablation experiment on the OTB-100 dataset.

To further validate the correctness of the ablation study results obtained on the GOT-10k dataset, this study extended the experimental scope to the OTB-100 dataset. The ablation study on the OTB-100 aimed to further verify the specific tracking effects demonstrated in the ablation study. The results are detailed in Table 2, with the success rate curves and precision rate curves depicted in Figure 8 and Figure 9, respectively.

The experimental outcomes on the OTB-100 dataset corroborate the conclusions drawn from the GOT-10k dataset ablation study. The results demonstrate that the accuracy and precision of tracking were improved with the gradual integration of various enhancements in the baseline TransT model. Notably, when all enhancements were integrated into the TransT model, forming the improved proposed PCA-T model, the model achieved a success rate of 0.696 and a precision rate of 0.910, surpassing the performance of the other models in the ablation study. These results fully substantiate the effectiveness of the proposed method.

### 4.3. Quantitative Analysis

To thoroughly assess the performance of the proposed PCA-T model, this study conducted a comparative analysis between the PCA-T model and five contemporary models within the field of object tracking, including the transformer-based TransT model; the Siamese network-based SiamBC, SiamCAR, and SiamRPN models; and the correlation filter-based Staple model. The quantitative evaluation of the models was performed using the GOT-10k and OTB-100 datasets.

**Figure 10:**
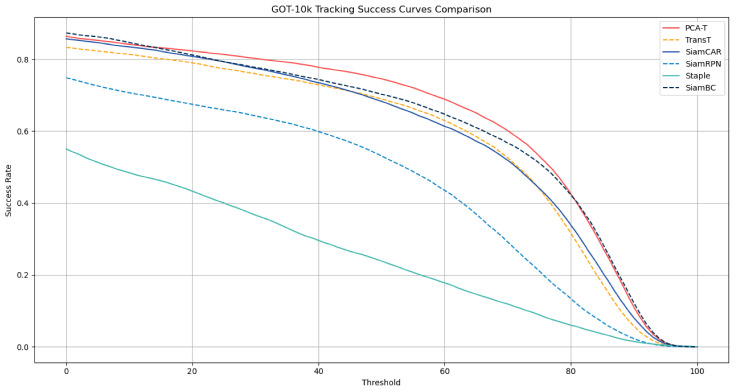
Success rate curves of 6 tracking models on the GOT-10k dataset.

For the assessment of model performance on the GOT-10k dataset, this study employed three metrics: average overlap (AO), success rate (SR), and success rate curve (SRC). The average overlap refers to the mean of the overlap ratios between the target region and the ground truth across all frames. The success rate is the proportion of frames where the overlap ratio exceeds a certain threshold (e.g., 0.5 or 0.75) relative to the total number of frames. The success rate curve illustrates the variation in the success rate at different thresholds. The results of the PCA-T model in terms of average overlap and success rate compared to those of the other methods are presented in Table 3, while the results of the success rate curves are depicted in Figure 10.

The experimental results indicate that the enhanced PCA-T model outperformed the comparative models across three evaluation metrics: average overlap (AO), success rate (SR), and success rate curve (SRC). The model maintained a high success rate across various thresholds, demonstrating its robustness and accuracy.

**Figure 11:**
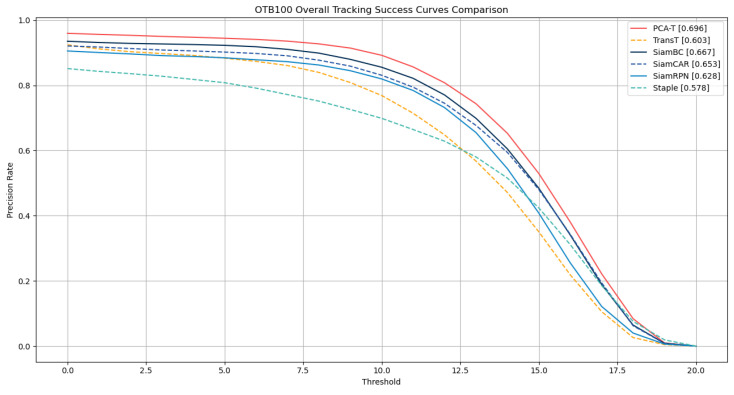
Success rate curves of 6 tracking models on the OTB-100 dataset.

**Figure 12:**
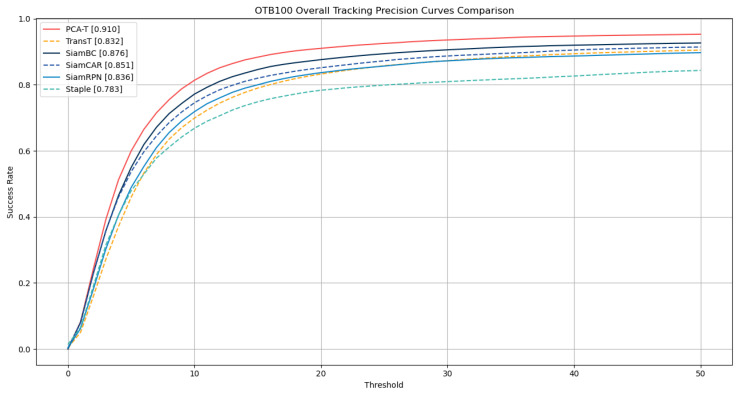
The precision rate curves of 6 tracking models on the OTB-100 dataset.

On the OTB-100 dataset, this study employed a one-pass evaluation (OPE) approach to quantify the performance of the tracking algorithms, selecting the success rate (SR) and precision rate (PR) as the metrics for assessment. The specific numerical results are summarized in Table 4, while the success rate curves and precision rate curves are presented in Figure 11 and Figure 12, respectively.

The experimental results on the OTB-100 dataset demonstrate that the proposed PCA-T model outperformed five existing models in both the success rate and precision rate, further confirming the superior performance and significant advantages of the PCA-T model in addressing object tracking tasks.

Based on a comprehensive analysis of the experimental results on the OTB-100 dataset, this study further explored the specific performance of the proposed PCA-T model under various complex scenarios. Utilizing the 11 video attributes of the OTB-100 dataset, which include illumination changes, scale variations, occlusions, motion blur, and seven other distinct scene attributes, this study categorized these attributes and calculated the accuracy and precision performance of each model within each category. The success rate data are summarized in Table 5, while the precision rate data are presented in Table 6.

The experimental results revealed that the PCA-T model achieved exceptional tracking performance across multiple video attributes. Specifically, the PCA-T model achieved the best success rate performance in ten video attributes, including illumination variation (IV), scale variation (SV), occlusion (OCC), deformation (DEF), fast motion (FM), in-plane rotation (IPR), out-of-plane rotation (OPR), out-of-view (OV), background clutter (BC), and low resolution (LR). In the motion blur (MB) scenario, although the PCA-T model slightly underperformed compared to the SiamCAR model, the gap between the two was merely 0.014, indicating that the PCA-T model still maintained a high level of performance in this scenario.

In terms of precision, the PCA-T model demonstrated the best performance in scenarios such as illumination variation (IV), occlusion (OCC), background clutter (BC), and low resolution (LR), and also exhibited strong performance in scale variation (SV), deformation (DEF), in-plane rotation (IPR), and out-of-plane rotation (OPR), with data differences from the optimal model not exceeding 0.03. Although the PCA-T model’s performance was somewhat lacking in the fast motion (FM), motion blur (MB), and out-of-view (OV) scenarios, the overall gap from the optimal model remained less than 0.09.

These results indicate that the PCA-T model has a significant advantage in terms of the success rate and is capable of maintaining accurate target tracking and localization under various complex conditions, especially in challenging scenarios, such as illumination variation, occlusion, background clutter, and low resolution, where its performance was particularly outstanding. Moreover, despite a slight decrease in precision in certain extreme conditions, the overall precision performance of the PCA-T model remained good, indicating the model’s effectiveness in controlling the center error. Therefore, based on these results, it can be concluded that the PCA-T model demonstrates strong performance and broad applicability in video tracking tasks, making it a reliable choice with high success and precision rates in a variety of video tracking tasks.

In summary, the proposed PCA-T model underwent a rigorous quantitative evaluation of its performance in the field of object tracking and was validated on the widely recognized GOT-10k and OTB-100 datasets. The evaluation results showed that the PCA-T model outperformed comparative models in several key performance indicators, including the average overlap (AO), success rate (SR), and precision rate (PR), thereby achieving superior tracking performance.

Furthermore, a detailed attribute classification and performance comparison analysis of the video sequences in the OTB-100 dataset further confirmed the outstanding performance of the PCA-T model across a diverse range of tracking scenarios. Particularly in the handling of complex scenarios such as illumination variation, scale variation, and occlusion, the model showed significant robustness and accuracy. Although the performance of the PCA-T model in the motion blur scenario was slightly lower than that of the SiamCAR model, the overall performance of the PCA-T model still surpassed that of the comparative models in other scenarios.

Considering all factors, the overall performance advantages of the PCA-T model in object tracking tasks, particularly its robustness and accuracy in complex video scenarios, demonstrate its potential and value in practical applications. These findings provide new perspectives for the development of object tracking technology and lay a solid foundation for future research directions and application practices.

### 4.4. Qualitative Inorganic Analysis

In order to thoroughly investigate the performance of the proposed PCA-T model in various challenging and complex scenarios, we selected four representative video sequences from the OTB-100 dataset for keyframe tracking tests and conducted a comparative analysis with other tracking models, including TransT, SiamBC, SiamCAR, SiamRPN, and Staple.

**Figure 13:**
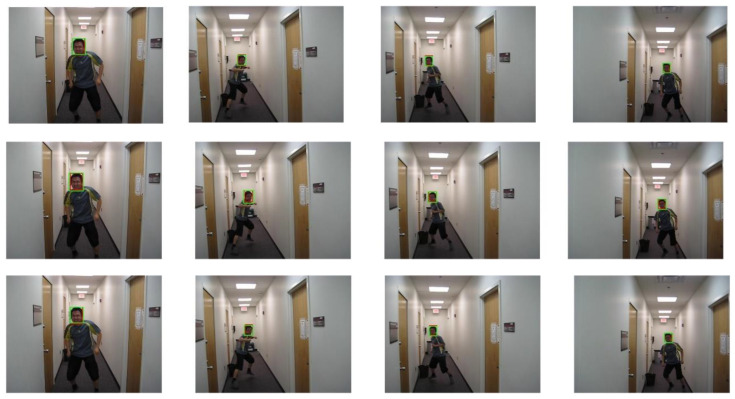
Test results of the PCA-T, TransT, and SiamBC models for the boy sequence.

**Figure 14:**
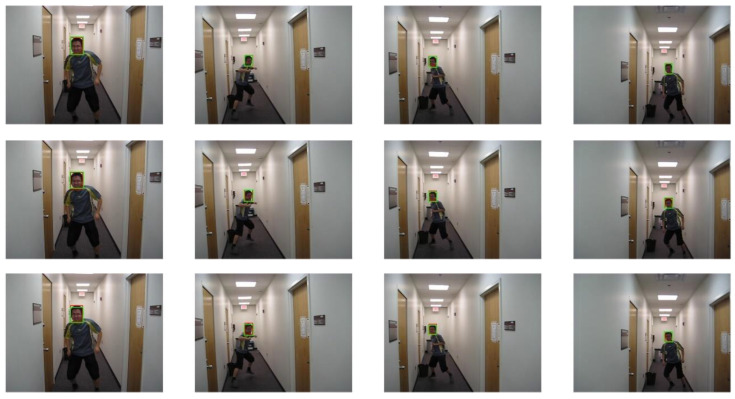
Test results of the SiamCAR, SiamRPN, and Staple models for the boy sequence.

**Figure 15:**
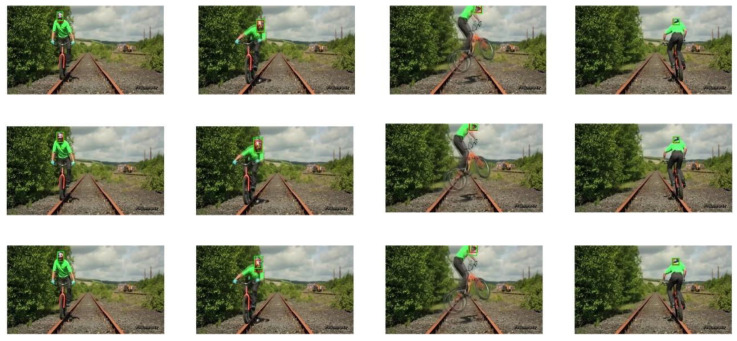
Test results of the PCA-T, TransT, and SiamBC models for the biker sequence.

**Figure 16:**
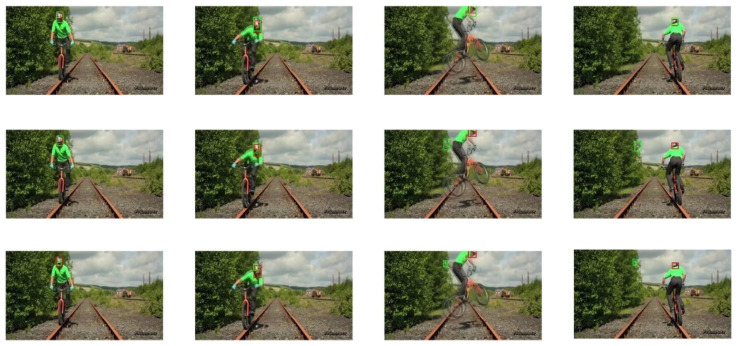
Test results of the SiamCAR, SiamRPN, and Staple models for the biker sequence.

**Figure 17:**
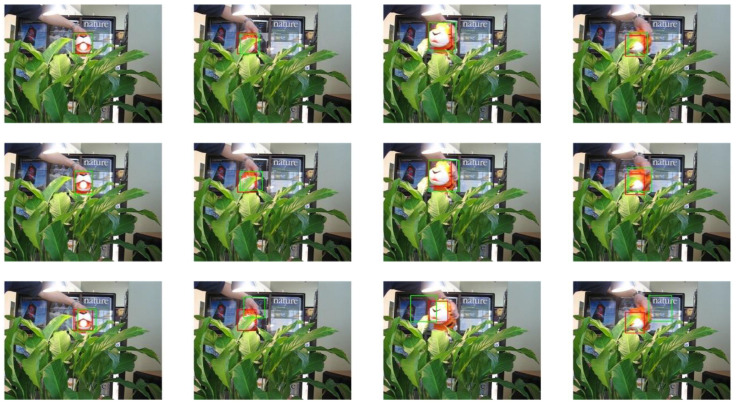
Test results of the PCA-T, TransT, and SiamBC models for the tiger1 sequence.

**Figure 18:**
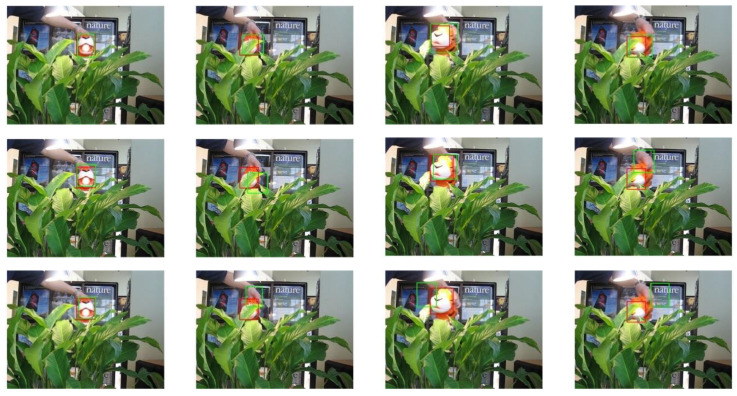
Test results of the SiamCAR, SiamRPN, and Staple models for the tiger1 sequence.

**Figure 19:**
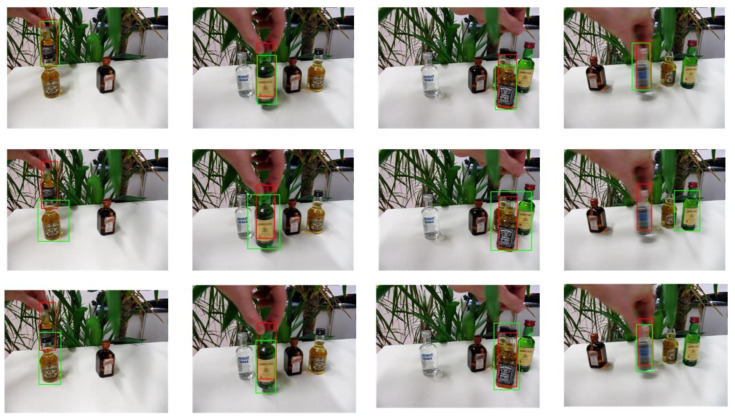
Test results of the PCA-T, TransT, and SiamBC models for the liquor sequence.

**Figure 20:**
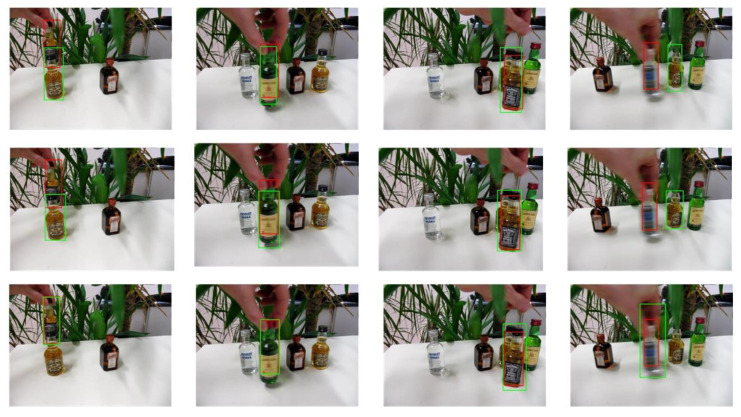
Test results of the SiamCAR, SiamRPN, and Staple models for the liquor sequence.

The selected video sequences comprehensively cover typical tracking challenges, including illumination variations, scale changes, occlusions, deformations, and motion blur. These challenges were integrated into the tracking process to fully test the robustness of the model. Table 7 provides a detailed list of the attributes and characteristics of the selected video sequences, while Figure 13, Figure 14, Figure 15, Figure 16, Figure 17, Figure 18, Figure 19 and Figure 20 present the tracking effects of each model for the selected video sequences through intuitive visualization, offering a visual reference for subsequent performance comparison and analysis.

In the experimental study of the “boy” video sequence, the keyframes at frames 320, 345, 397, and 602 were chosen for in-depth analysis. As depicted in Figure 13 and Figure 14, the target object underwent in-plane rotation in frames 320 and 345. Frame 397 featured an out-of-plane rotation due to a change in the camera’s perspective, and in frame 602, a change in the camera distance led to a scale variation in the target object. The experimental results indicate that the proposed PCA-T model demonstrated excellent tracking accuracy on the keyframes of the “boy” video sequence, effectively handling complex scenarios involving multiple attribute combinations such as scale changes, in-plane rotations, and out-of-plane rotations. This further confirms the model’s reliability in these specific scenarios and showcases its high efficiency in object tracking tasks.

In the in-depth analysis of the “biker” video sequence, the keyframes at frames 42, 65, 74, and 90 were meticulously examined. As shown in Figure 15 and Figure 16, the target experienced rapid motion in frames 65 and 74, resulting in significant motion blur. Additionally, in frames 74 and 90, the target underwent shape changes and rotations. The experimental results demonstrate that the PCA-T model proposed in this study can accurately locate and track the target object’s size changes even in challenging tracking situations such as rapid motion, motion blur, and rotation. In comparison, the comparative tracking models exhibited varying degrees of tracking drift and scale estimation errors in these scenarios. This finding further corroborates the significant effectiveness of the PCA-T model in addressing high-difficulty tracking tasks, such as rapid motion and motion blur.

In the experimental study of the “tiger1” video sequence, the keyframes at frames 31, 60, 90, and 215 were selected for in-depth comparative analysis. As illustrated in Figure 17 and Figure 18, the target’s mouth-opening and -closing movements in frames 31 and 90, along with the introduction of a new light source, led to significant changes in lighting conditions, which had a substantial impact on the target’s appearance features. In frames 60 and 215, parts of the target were occluded by vegetation, challenging its integrity. The experimental results show that the proposed tracking model was able to effectively stabilize the tracking of the target object’s position and size, while the other four tracking models exhibited varying degrees of drift or scaling errors. This phenomenon further confirms the superiority of the proposed model in handling complex scenarios such as deformations, lighting changes, and occlusions. The robust performance of the model provides an effective solution for object tracking tasks in video sequences, particularly when faced with changes in the target’s appearance and environmental interference.

In the experimental study of the “liquor” video sequence, the keyframes at frames 360, 728, 890, and 1504 were selected for in-depth comparative analysis. As shown in Figure 19 and Figure 20, background clutter was particularly prominent in frames 360 and 890, mainly due to the high color similarity and partial overlap between the tracking target and background objects. In frames 728 and 1504, the tracking target encountered significant occlusions and even completely left the field of view at certain moments, further increasing the complexity of the tracking task. Nevertheless, the PCA-T model exhibited excellent performance in handling these complex scenarios. Compared to other deep learning models, the PCA-T model was more effective in dealing with background clutter and situations where the target left the field of view. Although there were inevitable errors during the tracking process, overall, the model successfully completed the continuous tracking of the target. This result not only validates the effectiveness of the PCA-T model in dealing with such complex video sequences but also provides strong theoretical support and practical guidance for future applications in similar scenarios.

In summary, this study conducted a comprehensive performance evaluation of the PCA-T model by comparing it with five top tracking models and verifying its tracking capabilities in four video sequences (boy, biker, tiger1, and liquor) from the OTB-100 dataset. The experimental results show that the PCA-T model possesses significant performance advantages in handling various tracking challenges, such as illumination changes, scale changes, occlusions, deformations, and motion blur. Especially in keyframe analysis, the model can more accurately track the target object, maintaining stable tracking effects even when the target’s appearance features undergo significant changes or the camera conditions change drastically.

## 5. Conclusions

In this study, a novel object tracking method is proposed to address the limitations of existing models in feature extraction. By introducing a pyramid channel attention mechanism, the method’s adaptability to variations in object size and shape is enhanced, thereby significantly improving the discriminatory power and expressive capability of features. Furthermore, this paper incorporates a hierarchical cross-attention mechanism and an adaptive multi-layer perceptron (aMLP) to construct a hierarchical network architecture. These innovations, combined with nonlinear transformations, enable more precise matching of object features and more efficient optimization of information flow. The experimental results demonstrate the superior performance of the proposed method compared to other state-of-the-art models, especially in complex scenarios.

This research holds significant implications and value for the development of various fields, including robotic navigation, autonomous driving, intelligent surveillance, and human–computer interaction. The enhanced tracking accuracy and robustness provided by the method proposed in this paper can directly contribute to the advancement of these applications, making systems more reliable and efficient.

Looking ahead, our future research efforts in object tracking technology will focus on further enhancing the generalization, real-time performance, and universality of algorithms to meet the growing demands of practical applications. By continuously improving these aspects, we aim to drive the development of more intelligent, efficient, and reliable object tracking solutions that can be widely applied across diverse fields.

## Figures and Tables

**Table 1:** Ablation experiment results.

Model Configuration	Pyramid Channel Attention	Hierarchical Cross-Attention	aMLP	AO	SR_0.5_	SR_0.75_
Baseline	No	No	No	0.572	0.690	0.440
Modification 1	Yes	No	No	0.593	0.711	0.476
Modification 2	Yes	Yes	No	0.619	0.723	0.527
Modification 3	Yes	Yes	Yes	0.625	0.746	0.530

**Table 2:** Ablation experiment results on the OTB-100 dataset.

Model Configuration	Pyramid Channel Attention	Hierarchical Cross-Attention	aMLP	Success	Precision
Baseline	No	No	No	0.603	0.832
Modification 1	Yes	No	No	0.645	0.848
Modification 2	Yes	Yes	No	0.691	0.900
Modification 3	Yes	Yes	Yes	0.696	0.911

**Table 3:** Experimental results of 6 tracking models on the GOT-10k dataset.

Model	AO	SR_0.5_	SR_0.75_
PCA-T	0.625	0.746	0.530
TransT	0.572	0.690	0.440
SiamBC	0.607	0.703	0.511
SiamCAR	0.581	0.683	0.441
SiamRPN	0.441	0.531	0.221
Staple	0.246	0.239	0.089

**Table 4:** Experimental results of 6 tracking models on the OTB-100 dataset.

Model	Success	Precision
PCA-T	0.696	0.910
Trans	0.572	0.690
SiamBC	0.667	0.876
SiamCAR	0.653	0.851
SiamRPN	0.628	0.836
Staple	0.578	0.783

**Table 5:** Success rates of 6 tracking models on 11 attributes of the OTB-100 dataset.

Attribute	PCA-T	TransT	SiamBC	SiamCAR	SiamRPN	Stapled
IV	0.724	0.585	0.676	0.657	0.614	0.616
SV	0.693	0.608	0.668	0.676	0.629	0.562
OCC	0.648	0.570	0.630	0.596	0.562	0.551
DEF	0.662	0.580	0.636	0.608	0.589	0.558
MB	0.698	0.619	0.682	0.712	0.634	0.538
FM	0.687	0.612	0.669	0.682	0.626	0.553
IPR	0.718	0.630	0.660	0.664	0.661	0.568
OPR	0.687	0.593	0.651	0.642	0.617	0.555
OV	0.639	0.529	0.600	0.638	0.574	0.483
BC	0.680	0.500	0.654	0.578	0.595	0.602
LR	0.719	0.633	0.640	0.693	0.632	0.528

**Table 6:** Precision rates of 6 tracking models on 11 attributes of the OTB-100 dataset.

Attribute	PCA-T	TransT	SiamBC	SiamCAR	SiamRPN	Staple
IV	0.857	0.804	0.800	0.824	0.815	0.734
SV	0.827	0.835	0.801	0.835	0.832	0.690
OCC	0.786	0.782	0.765	0.777	0.741	0.610
DEF	0.812	0.820	0.780	0.820	0.787	0.685
MB	0.818	0.814	0.798	0.901	0.833	0.621
FM	0.817	0.812	0.788	0.876	0.878	0.643
IPR	0.864	0.867	0.802	0.876	0.894	0.703
OPR	0.832	0.836	0.795	0.857	0.830	0.685
OV	0.785	0.721	0.711	0.832	0.736	0.571
BC	0.816	0.680	0.792	0.758	0.808	0.728
LR	0.944	0.897	0.842	0.931	0.873	0.746

**Table 7:** Video sequences and corresponding challenge attributes.

Video Sequence	Resolution	Frame Count	Challenge Attributes
boy	640 × 480	602	SV, MB, FM, IPR, OPR
biker	640 × 360	142	SV, OCC, MB, FM, OPR, LR
tiger1	640 × 480	354	IV, OCC, DEF, MB, FM, IPR, OPR
liquor	640 × 480	1741	IV, SV, OCC, MB, FM, OPR, OV, BC

## Data Availability

Data will be made available on request.
